# A New Strategy for a High Tumour Burden Region: The Campania Oncological Network

**Published:** 2020-02-20

**Authors:** S De Placido, U Bracale, R Pacelli, M Sodo, G Merola, V Silvestri, F Corcione

**Affiliations:** 1 University Hospital School of Medicine Federico II of Naples, Department of Clinical Medicine and Surgery; 2 University Hospital School of Medicine Federico II of Naples, Department of Gastroenterology, Endocrinology and Surgical Endoscopy; 3 University Hospital School of Medicine Federico II of Naples, Department of Advanced Biomedical Sciences; 4 University Hospital School of Medicine Federico II of Naples, Vascular and Endovascular Surgery Unit, Department of Public Health

In the period from 2006 to 2019 the Italian Association of Medical Oncology (AIOM) reported a tumour burden with a prevalence in cancer cases rising from 225,000 to 346,000 1. One of the major issues of oncological disease in Italy today is its geographical distribution, with an increasing incidence disparity from north (4%) to south (14%).

For instance, today in Italy 63% of women and 54% of men with colon cancer are alive 5 years after diagnosis however, if assessed as a whole, Italy’s survival rate for colon cancer is equal to or higher than the European average. However, these statistics are not homogenous amongst all region as the southern Italian regions report a lower survival rate than the rest of the country.

This data may not only be due to different lifestyle habits and educational and cultural levels throughout Italy, but perhaps also to adherence to locally available screening programs by southern Italian patients. The Italian National Institute of Health (INIH) reports that each year in the southern Italian region of Campania (consisting of Naples, the third largest city in Italy, Salerno and the provincial capitals of Caserta, Avellino, Benevento and Nola) 398 new cases of cancer per 100,000 inhabitants were reported for the male sex versus a national rate of 336 per 100,000 inhabitants 1–2. An analysis of the number of surgical procedures for the first three most frequent oncological diseases (colorectal, breast and prostate cancer) reveals that almost 7000 major procedures were performed in the year 2016 in the Campania Region. Despite the impressive number of patients, and the fully technologically equipped hospitals available, over the last ten years a notable “health migration” to access northern Italian health care facilities was prevalent.

The ARSAN (Regional Agency for Health), during the 2007–2013 period, registered a high rate of “health migration” from the Campania Region to northern regions for the cure of patients affected, for example, with colon cancer. In the district of Caserta, 44–45% of colo-rectal cancer procedures diagnosed locally were performed outside the region. Moreover, during the year 2012, 7 online cancer registries were set up in Campania, although today only three are currently working.

In this scenario, three causes have been identified:

- Low compliance to screening programs
- Difficulties in accessing hospitals
- Division of diagnostic and therapeutic pathways between hospitals

Based upon these issues the Campania Region Health Committee decided to set up an oncological network (CON) based on the latest evidence of best practices for oncology. In particular, the first aim of the network was to properly channel the flow of patients for colorectal, breast and uterine cancer 3 with the intent of keeping them in the region and not seeking recourse elsewhere.

The oncological network is based on four pillars: Epidemiology, Guidelines and Screening, Qualified Hospitals and Control of Outcomes.

Regarding the previous outcome criteria and problems of the various regional hospitals, CON identified which hospitals had the most volume of diagnostic and therapeutic procedures for the aforementioned cancers. Based upon this the CON then directed all patients in the regional hospitals with the best-experienced or most qualified health professionals.

With the passing of Regional Act n°98 in 2016, the Campania Oncological Network (CON) was founded.

The major aims of CON are the establishment of:

1. Welfare Diagnostic and Therapeutic Pathways (WDTP)
  and
2. Multidisciplinary Oncological Groups (MOGs)

This model encompasses all the regional health frameworks (Prevention, Diagnosis, Care and Rehabilitation) and was inspired by the National Comprehensive Cancer Care Network.

The WDTP is an internal guideline for the diagnosis and care that the patient has to follow in order to access the best standards of care for each type of disease. For example, in order to achieve the best surgical outcome for colo-rectal cancer, the hospital must provide the possibility for surgical treatment with a laparoscopic approach or less invasive procedures 4–7.

The second aspect of quality assessment is based on outcome control by external health inspectors/examiners, basing their evaluations on performance indicators for costs, clinical management and outcomes.

More generally, CON is a network model that aims to:

- develop and share practical guidelines
- share staff training
-exchange health information (ex, medical registries, information flows, etc.)
- provide a standard rating system

Moreover, CON is built upon four different competence levels as reported below:

- First Level Center (Screening Center)
- Second Level Center or Multidisciplinary Referral Oncological Center: CORP (diagnostic, therapeutic and follow up functions)
- Third Level Center: CORPUS, with specific oncological activities.
- Hospice and Outpatient Clinics for pain Management.

CORP centers are a cluster of different clinic units with a common health care aim and are identified by different parameters as well as by surgical activities.

Some of these parameters include: > 200 cases/year during 2013–2015 for colorectal, breast and cervical cancer, the concurrent presence of a General Surgical Unit, Oncological Unit and Specialist Units (Radiotherapy, Pathological Anatomy, Radiology), Scientific and training benchmarks

CORPUS’ purpose is that of higher research and training functions.

In conclusion by optimizing specific pathways for oncological patients and selecting/channelling patients to the appropriate highly specialized centers, CON aims to improve oncological outcomes in Campania while also reducing health migration to other Italian regions for treatment^8^. The newly established mission of the Campania Regional Health System is to guarantee to the local oncological user a level of quality assistance that includes equity of access, clinical and organizational appropriateness of care and settings and health structure integration for the successful completion of treatment courses and procedural quality-control.
